# Adaptive Unscented Kalman Filter for Target Tracking with Unknown Time-Varying Noise Covariance

**DOI:** 10.3390/s19061371

**Published:** 2019-03-19

**Authors:** Baoshuang Ge, Hai Zhang, Liuyang Jiang, Zheng Li, Maaz Mohammed Butt

**Affiliations:** 1 School of Automation Science and Electrical Engineering, Beihang University, No. 37 Xueyuan Road, Haidian District, Beijing 100083, China; gebaoshuang@buaa.edu.cn (B.G.); j279795967@126.com (L.J.); lizheng@buaa.edu.cn (Z.L.); maazmb@gmail.com (M.M.B.); 2 Science and Technology on Aircraft Control Laboratory, Beihang University, No. 37 Xueyuan Road, Haidian District, Beijing 100083, China

**Keywords:** adaptive filtering, data fusion, target tracking, non-linear filtering, unknown noise statistics

## Abstract

The unscented Kalman filter (UKF) is widely used to address the nonlinear problems in target tracking. However, this standard UKF shows unstable performance whenever the noise covariance mismatches. Furthermore, in consideration of the deficiencies of the current adaptive UKF algorithm, this paper proposes a new adaptive UKF scheme for the time-varying noise covariance problems. First of all, the cross-correlation between the innovation and residual sequences is given and proven. On this basis, a linear matrix equation deduced from the innovation and residual sequences is applied to resolve the process noise covariance in real time. Using the redundant measurements, an improved measurement-based adaptive Kalman filtering algorithm is applied to estimate the measurement noise covariance, which is entirely immune to the state estimation. The results of the simulation indicate that under the condition of time-varying noise covariances, the proposed adaptive UKF outperforms the standard UKF and the current adaptive UKF algorithm, hence improving tracking accuracy and stability.

## 1. Introduction

The main mission of target tracking is to estimate the dynamic parameters and show the trajectory of a maneuvering target by extracting the useful information from sensor observations [1]. Target tracking has a wide variety of both military and civilian applications in fields such as precision guidance, target recognition, and surveillance [2,3,4,5,6]. To implement target tracking in these research areas, filtering is being used increasingly in more recent tracking systems. Therefore, as a result, the tracking accuracy is to a large extent determined by the performance of the filter [7]. Although the statistical properties of measurement noise can be obtained in advance from the tracking sensor’s physical characteristics, these aren’t reliable, since they are affected by the external interference, especially in complicated environments. In addition, it is difficult to obtain the system noise with an accurate statistical covariance because of the random characteristics of acceleration and external manipulation [8]. The time-varying noise covariances involved in the maneuvering target tracking system make the traditional non-adaptive filtering algorithms no longer suitable. Therefore, an adaptive and stable filtering algorithm with high performance is required to deal with the noise covariance uncertainty.

The Kalman filter is one of the best-known algorithms for dealing with the problem of state estimation. This filter is based on the criterion of minimum mean square error, which can provide the optimal estimation of a linear system by using knowledge of the exact statistics information of the system parameters and the measurements [9]. Nonetheless, the nonlinearity of the tracking system and the time-varying noise statistic characteristics limit the applications of the Kalman filter. The extended Kalman filter (EKF) linearizes the non-linear models using Taylor series expansions to make them suit the Kalman filter algorithm [10,11]. However, the unknown and time-varying noise covariance makes EKF limited in the field of target tracking applications. To achieve accurate location estimation for a maneuvering target, adaptive EKF algorithms have been developed [12,13] that can update the noise covariance during the estimation process. Despite the covariance estimation by adaptive EKF, under strong nonlinearity, the basic drawback of EKF still exists, wherein the series approximations often lead to poor representation of the posterior density distribution of the state. The generic particle filter (PF), also known as sequential Monte Carlo estimation, can deal with nonlinearities of target tracking problem by evaluating the posterior density distribution with a large number of particles; however, this requires high computation cost and also has a well-known problem with respect to sample impoverishment [14,15]. 

Instead of linearization, the unscented Kalman filter (UKF) uses unscented transform to evaluate the nonlinear propagation of the state error covariance by producing a minimal special set of sigma points [16,17]. Through the carefully chosen sigma points, the accuracy of the posterior mean and covariance can be achieved to the third order for any Gaussian and nonlinear systems [1]. Overall, for nonlinear problems, the filtering precision of UKF is higher than EKF, and the computation complexity of UKF is less than that of PF [18,19]. Although UKF has sufficient advantages for the filtering problem of nonlinear systems, it becomes inaccurate and divergent in cases where the noise characteristics are unknown a priori. The interacting multiple model (IMM) algorithm is a well-known approach to target tracking, and provides a state estimate by giving weights to a combination of different model probabilities and the model switching probabilities. Therefore, this method can adapt to the uncertain noise of target motion-mode [20,21]. Although the integration of IMM algorithm and UKF can meet the challenges of noise uncertainty and system nonlinearity in maneuvering target tracking, the computational cost of this integration needs to be considered [22,23]. 

To deal with adaptive estimation of the noise covariance, many researchers have resorted to adaptive UKF algorithms. Zhang introduced the Sage-Husa statistics estimator to UKF in cases where the noise covariances are unknown, hence improving the accuracy of state estimation [24]. Soken and Hajiyev considered the covariance matching technique as a foundation, and put forward an adaptive fading UKF to accomplish the picosatellite attitude estimation in cases where process noise covariance changes [25]. However, these adaptation schemes may fail when the process and measurement noise covariances change at the same time. Furthermore, Li and Meng et al. proposed an adaptive UKF with noise statistic estimator that applies the innovation and residual sequences to estimate the covariance of the process and measurement noise [26,27]. Although these methods enhanced the adaptive capability of the standard UKF, one of their main deficiencies was that the determination of the process noise covariance was based on the orthogonality of innovation and residual sequences. Theoretically, the innovation and residual sequences extracted from the filter are correlative. Moreover, we can similarly see from the commonly used estimation methods proposed in [28,29,30] that the estimated method for measurement noise statistics is innovation-based and will be influenced by the state. Therefore, the estimation error will result in a risk of degradation in filter performance during the calculation of the measurement noise covariance. Aiming for this problem, Zhang et al. developed a measurement-based adaptive Kalman filtering algorithm (MAKF) that overcame the instability issue of improved Sage-Husa adaptive filter for the integrated navigation system [31,32]. Nonetheless, the MAKF is valid only if one of the measurement noise covariances is relatively smaller than the other, so that it can be neglected. To extend MAKF to any redundant measurement systems, an improved MAKF named redundant measurement noise covariance estimation (RMNCE) is proposed in [33,34], which is not only immune to the system state estimation error, but can also estimate the noise variance of the redundant measurement. 

In consideration of the deficiency of the algorithms mentioned above, and taking the advantages of the RMNCE algorithm, in this paper, a new adaptive UKF method is developed for nonlinear tracking systems with unknown time-varying noise covariance. The new algorithm avoids the interaction between the two types of noise and can estimate the covariance of the process and measurement noise simultaneously. In the proposed adaptive scheme, a new method of Q-estimation is deduced based on the correct correlation of the innovation and residual sequences. For the R-estimation, measurement-based noise covariance estimation is introduced, which avoids the negative influence of the inaccurate state estimation. Finally, the simulation results demonstrate that the proposed scheme can increase the tracking precision primarily because the estimated noise covariances are in accord with those of the real-time situations.

## 2. The UKF Algorithm for Nonlinear State Estimation 

Considering a general nonlinear discrete-time dynamic system, the process and measurement models can be described as follows

(1) $$\left\{ \begin{array}{l} X_{k}=f\left(X_{k-1}\right)+\Gamma_{k-1}W_{k-1}\\ Z_{k}=h\left(X_{k}\right)+V_{k} \end{array}\right.$$

where $X_{k}\in {\mathbb{R}}^{n\times 1}$ is the state vector, $Z_{k}\in {\mathbb{R}}^{m\times 1}$ denotes the measurement vector, f(⋅) and h(⋅) represent the known nonlinear state transition and measurement function, respectively. $\Gamma_{k-1}$ is the system noise-driven matrix. $W_{k-1}$ and $V_{k}$ are uncorrelated zero-mean Gaussian white noises whose covariances are $Q_{k-1}$ and $R_{k}$, respectively.

### 2.1. Standard UKF

The UKF algorithm is based on the notion that it should be easier to estimate a nonlinear distribution than to make an approximation of a nonlinear function [16]. In the standard UKF, the unscented transform is implemented to generate the sigma points to undergo the nonlinear transformation and calculate the first two moments of the transformed set. The general structure of the standard UKF algorithm can be described as follows:

Step 1: Initialization.

(2) $$\left\{ \begin{array}{l} {\hat{X}}_{0}=E\left\{X_{0}\right\}\\ P_{0}=E\left\{\left(X_{0}-{\hat{X}}_{0}\right){\left(X_{0}-{\hat{X}}_{0}\right)}^{T}\right\} \end{array}\right.$$

where ${\hat{X}}_{0}$ is the initial state and $P_{0}$ is the initial estimation error covariance.

Step 2: Sigma points calculation.

(3) $$\begin{array}{l} \chi_{k-1}^{\left(0\right)}={\hat{X}}_{k-1}\\ \chi_{k-1}^{\left(i\right)}={\hat{X}}_{k-1}+{\sqrt{\left(n+\lambda \right)P_{k-1}}}_{i},i=1,2,\cdots ,n\\ \chi_{k-1}^{\left(i\right)}={\hat{X}}_{k-1}-{\sqrt{\left(n+\lambda \right)P_{k-1}}}_{i},i=n+1,n+2,\cdots ,2n \end{array}$$

where *n* is the state dimension, *λ* = *α*^2^(*n* + *κ*) − *n* is the composite scaling factor. *α* and *κ* are tuning parameters. The parameter *α* is set to 0 ≤ *α* ≤ 1 and a good default setting on *κ* is *κ =* 0 [35].

Step 3: State prediction.

(4) $$\begin{array}{l} \chi_{k/k-1}^{\left(i\right)}=f\left(\chi_{k-1}^{\left(i\right)},k-1\right),i=0,1,2,\cdots ,2n\\ {\hat{X}}_{k/k-1}=\sum_{i=0}^{2n}\omega_{i}^{\left(m\right)}\chi_{k/k-1}^{\left(i\right)}\\ P_{XX}=\sum_{i=0}^{2n}\omega_{i}^{\left(c\right)}\left(\chi_{k/k-1}^{\left(i\right)}-{\hat{X}}_{k/k-1}\right){\left(\chi_{k/k-1}^{\left(i\right)}-{\hat{X}}_{k/k-1}\right)}^{T}\\ P_{k/k-1}=P_{XX}+\Gamma_{k-1}Q_{k-1}\Gamma_{k-1}^{T} \end{array}$$

where $\omega_{i}^{\left(m\right)}$ and $\omega_{i}^{\left(c\right)}$ are weights, which are defined as

(5) $$\begin{array}{l} \omega_{0}^{\left(m\right)}=\frac{\lambda }{n+\lambda }\\ \omega_{0}^{\left(c\right)}=\frac{\lambda }{n+\lambda }+\left(1-\alpha^{2}+\beta \right)\\ \omega_{i}^{\left(m\right)}=\omega_{i}^{\left(c\right)}=\frac{1}{2\left(n+\lambda \right)},i=1,2,\cdots ,2n \end{array}$$

where *β* ≥ 0 is introduced to incorporate the higher order information of the distribution, and the optimal setting is *β* = 2 for Gaussian distribution [36].

Step 4: Measurement prediction.

(6) $$\begin{array}{l} \zeta_{k/k-1}^{\left(i\right)}=h\left(\chi_{k/k-1}^{\left(i\right)},k\right),i=0,1,2,\cdots ,2n\\ {\hat{Z}}_{k/k-1}=\sum_{i=0}^{2n}\omega_{i}^{\left(m\right)}\zeta_{k/k-1}^{\left(i\right)}. \end{array}$$

Step 5: Kalman Gain calculation.

(7) $$\begin{array}{l} P_{XZ}=\sum_{i=0}^{2n}\omega_{i}^{\left(c\right)}\left(\chi_{k/k-1}^{\left(i\right)}-{\hat{X}}_{k/k-1}\right){\left(\zeta_{k/k-1}^{\left(i\right)}-{\hat{Z}}_{k/k-1}\right)}^{T}\\ P_{ZZ}=\sum_{i=0}^{2n}\omega_{i}^{\left(c\right)}\left(\zeta_{k/k-1}^{\left(i\right)}-{\hat{Z}}_{k/k-1}\right){\left(\zeta_{k/k-1}^{\left(i\right)}-{\hat{Z}}_{k/k-1}\right)}^{T}+R_{k}\\ K_{k}=P_{XZ}P_{ZZ}^{-1}. \end{array}$$

Step 6: Filtering update.

(8) $$\begin{array}{l} {\hat{X}}_{k}={\hat{X}}_{k/k-1}+K_{k}\left(Z_{k}-{\hat{Z}}_{k/k-1}\right)\\ P_{k}=P_{k/k-1}-K_{k}P_{ZZ}K_{k}^{T}. \end{array}$$

Step 7: For the next sample implement steps 2 to 6.

### 2.2. Problem Description of UKF for Time-Varying Noise Covariance

If the time-varying noise covariance is not correctly estimated in time, it will make the standard UKF algorithm inaccurate or divergent. Based on the steps of the standard UKF algorithm, it can be seen from Equation (4) that the calculation for the prediction covariance $P_{k/k-1}$ is influenced by the varying process noise covariance $Q_{k-1}$. Once the prediction covariance $P_{k/k-1}$ is contaminated, it will affect the estimation covariance $P_{k}$ via Equation (8) and then contaminate the sigma-point distribution at the next epoch. Finally, the incorrect mean and covariance derived from the contaminated distribution reduces the filtering accuracy. Moreover, the varying measurement noise covariance $R_{k}$ directly affects the calculation results of the filtering gain through Equation (7), hence making the standard UKF algorithm unstable. Although a few adaptive UKF algorithms were proposed in [25,27], these algorithms have flaws in estimating the process noise covariance. Hence, it is necessary to design an effective adaptive UKF algorithm for target tracking systems with unknown time-varying noise covariance. 

## 3. An Innovative Adaptive UKF Scheme

In this section, an innovative adaptive UKF scheme is developed, which makes optimal use of the information in the filtering process. The innovation and residual sequences are applied to estimate the process noise covariance ***Q*** and the redundant measurement difference sequences are exploited to estimate the measurement noise covariance ***R***. 

### 3.1. Adaptive **Q** Estimation

In Kalman filtering theory, the innovation $\varepsilon_{k}$ and the residual $\eta_{k}$ are defined according to [27,37] as

(9) $$\begin{array}{l} \varepsilon_{k}=Z_{k}-h\left({\hat{X}}_{k/k-1}\right)\\ \eta_{k}=Z_{k}-h\left({\hat{X}}_{k}\right) \end{array}$$

**Theorem.**  For a given system as described by Equation (1), the cross-correlation between the innovation and the residual at time *k* is

(10) $$E\left(\varepsilon_{k}\eta_{k}^{T}\right)=H_{k/k-1}P_{k}H_{k}^{T}+R_{k}\left(I-K_{k}^{T}H_{k}^{T}\right)$$

where $H_{k/k-1}={\frac{\delta h}{\delta X}|}_{{\hat{X}}_{k/k-1}}$ and $H_{k}={\frac{\delta h}{\delta X}|}_{{\hat{X}}_{k}}$ are Jacobian matrices at ${\hat{X}}_{k/k-1}$ and ${\hat{X}}_{k}$, respectively.

**Proof.**  Substitute the filtering update equations in Equation (8) into Equation (9) and evaluate the partial derivative matrix at the predicted state ${\hat{X}}_{k/k-1}$, then the residual can be rewritten as

(11) $$\begin{array}{ll} \eta_{k} & =Z_{k}-h\left({\hat{X}}_{k/k-1}+K_{k}\varepsilon_{k}\right)\\ & =Z_{k}-h\left({\hat{X}}_{k/k-1}\right)-H_{k/k-1}K_{k}\varepsilon_{k}\\ & =\left(I-H_{k/k-1}K_{k}\right)\varepsilon_{k}. \end{array}$$

According to Equation (11), it can be obtained that the residual vector is a linear combination of the innovation vector. Thus, they are non-orthogonal.

Considering the partial derivatives of the measurement function, substitute the measurement equations in Equation (1) into Equation (9). The innovation and residual sequences can be described as

(12) $$\begin{array}{ll} \varepsilon_{k} & =h\left(X_{k}\right)-h\left({\hat{X}}_{k/k-1}\right)+V_{k}\\ & \approx H_{k/k-1}\left(X_{k}-{\hat{X}}_{k/k-1}\right)+V_{k}\\ & =H_{k/k-1}{\tilde{X}}_{k/k-1}+V_{k}\\ \eta_{k} & =h\left(X_{k}\right)-h\left({\hat{X}}_{k}\right)+V_{k}\\ & \approx H_{k}\left(X_{k}-{\hat{X}}_{k}\right)+V_{k}\\ & =H_{k}{\tilde{X}}_{k}+V_{k}. \end{array}$$

where ${\tilde{X}}_{k/k-1}$ denotes the prediction error and ${\tilde{X}}_{k}$ represents the estimation error.

According to Equation (12), the cross-correlation between the innovation and the residual at time *k* is expressed as

(13) $$\begin{array}{ll} E\left(\varepsilon_{k}\eta_{k}^{T}\right) & =E\left\{\left(H_{k/k-1}{\tilde{X}}_{k/k-1}+V_{k}\right){\left(H_{k}{\tilde{X}}_{k}+V_{k}\right)}^{T}\right\}\\ & =E\left\{H_{k/k-1}{\tilde{X}}_{k/k-1}{\tilde{X}}_{k}^{T}H_{k}^{T}+H_{k/k-1}{\tilde{X}}_{k/k-1}V_{k}^{T}+V_{k}{\tilde{X}}_{k}^{T}H_{k}^{T}+V_{k}V_{k}^{T}\right\}\\ & =H_{k/k-1}E\left\{{\tilde{X}}_{k/k-1}{\tilde{X}}_{k}^{T}\right\}H_{k}^{T}+H_{k/k-1}E\left\{{\tilde{X}}_{k/k-1}V_{k}^{T}\right\}+E\left\{V_{k}{\tilde{X}}_{k}^{T}\right\}H_{k}^{T}+E\left\{V_{k}V_{k}^{T}\right\}. \end{array}$$

Due to the assumption that the process and measurement noises are uncorrelated, we have $E\left\{{\tilde{X}}_{k/k-1}V_{k}^{T}\right\}=0$. The cross-correlation $E\left\{{\tilde{X}}_{k/k-1}{\tilde{X}}_{k}^{T}\right\}$ and $E\left\{V_{k}{\tilde{X}}_{k}^{T}\right\}$ can be written as follows

(14) $$\begin{array}{ll} E\left\{{\tilde{X}}_{k/k-1}{\tilde{X}}_{k}^{T}\right\} & =E\left\{{\tilde{X}}_{k/k-1}{\left[\left(I-K_{k}H_{k/k-1}\right){\tilde{X}}_{k/k-1}-K_{k}V_{k}\right]}^{T}\right\}\\ & =E\left\{{\tilde{X}}_{k/k-1}{\tilde{X}}_{k/k-1}^{T}{\left(I-K_{k}H_{k/k-1}\right)}^{T}-{\tilde{X}}_{k/k-1}V_{k}^{T}K_{k}^{T}\right\}\\ & =P_{k/k-1}{\left(I-K_{k}H_{k/k-1}\right)}^{T}\\ & =P_{k}^{T}=P_{k} \end{array}$$

(15) $$\begin{array}{ll} E\left\{V_{k}{\tilde{X}}_{k}^{T}\right\} & =E\left\{V_{k}{\left[\left(I-K_{k}H_{k/k-1}\right){\tilde{X}}_{k/k-1}-K_{k}V_{k}\right]}^{T}\right\}\\ & =E\left\{V_{k}{\tilde{X}}_{k/k-1}^{T}{\left(I-K_{k}H_{k/k-1}\right)}^{T}-V_{k}V_{k}^{T}K_{k}^{T}\right\}\\ & =-R_{k}K_{k}^{T} \end{array}$$

where $E\left\{{\tilde{X}}_{k/k-1}{\tilde{X}}_{k/k-1}^{T}\right\}=P_{k/k-1}$ and $E\left\{V_{k}V_{k}^{T}\right\}=R_{k}$.

Substitute Equations (14) and (15) back into Equation (13), the cross-correlation between the innovation and the residual at time *k* can be obtained as

(16) $$\begin{array}{ll} E\left(\varepsilon_{k}\eta_{k}^{T}\right) & =E\left\{\left(H_{k/k-1}{\tilde{X}}_{k/k-1}+V_{k}\right){\left(H_{k}{\tilde{X}}_{k}+V_{k}\right)}^{T}\right\}\\ & =H_{k/k-1}P_{k}H_{k}^{T}-R_{k}K_{k}^{T}H_{k}^{T}+R_{k}\\ & =H_{k/k-1}P_{k}H_{k}^{T}+R_{k}\left(I-K_{k}^{T}H_{k}^{T}\right). \end{array}$$

This completes the proof. □

**Remark.**  Considering the innovation and residual sequences are zero means, the covariance $\mathrm{Cov}\left(\varepsilon_{k},\eta_{k}^{}\right)$ is equal to $E\left(\varepsilon_{k}\eta_{k}^{T}\right)$. If the Jacobian matrices are evaluated at the same state, $E\left(\varepsilon_{k}\eta_{k}^{T}\right)$ is symmetric, and we have $E\left(\varepsilon_{k}\eta_{k}^{T}\right)=E\left(\eta_{k}\varepsilon_{k}^{T}\right)$. Otherwise, for a small sampling time, the Jacobian matrices $H_{k/k-1}\approx H_{k}$. Thus, $E\left(\varepsilon_{k}\eta_{k}^{T}\right)\approx E\left(\eta_{k}\varepsilon_{k}^{T}\right)$.

To improve the robustness of the ***Q***-estimation, both the innovations and the residuals are used [37]. Taking the expectation of the difference between innovation and residual follows that

(17) $$E\left[\left(\eta_{k}-\varepsilon_{k}\right){\left(\eta_{k}-\varepsilon_{k}\right)}^{T}\right]=E\left(\varepsilon_{k}\varepsilon_{k}^{T}\right)+E\left(\eta_{k}\eta_{k}^{T}\right)-E\left(\varepsilon_{k}\eta_{k}^{T}\right)-E\left(\eta_{k}\varepsilon_{k}^{T}\right).$$

From Equation (12), the innovation covariance $E\left(\varepsilon_{k}\varepsilon_{k}^{T}\right)$ can be written as

(18) $$\begin{array}{ll} E\left(\varepsilon_{k}\varepsilon_{k}^{T}\right) & =E\left\{\left(H_{k/k-1}{\tilde{X}}_{k/k-1}+V_{k}\right){\left(H_{k/k-1}{\tilde{X}}_{k/k-1}+V_{k}\right)}^{T}\right\}\\ & =H_{k/k-1}E\left\{{\tilde{X}}_{k/k-1}{\tilde{X}}_{k/k-1}^{T}\right\}H_{k/k-1}^{T}+E\left\{V_{k}V_{k}^{T}\right\}\\ & =H_{k/k-1}P_{k/k-1}H_{k/k-1}^{T}+R_{k}. \end{array}$$

Based on Equations (12) and (15), the residual covariance $E\left(\eta_{k}\eta_{k}^{T}\right)$ can be obtained as follows

(19) $$\begin{array}{ll} E\left(\eta_{k}\eta_{k}^{T}\right) & =E\left\{\left(H_{k}{\tilde{X}}_{k}+V_{k}\right){\left(H_{k}{\tilde{X}}_{k}+V_{k}\right)}^{T}\right\}\\ & =H_{k}E\left\{{\tilde{X}}_{k}{\tilde{X}}_{k}^{T}\right\}H_{k}^{T}+H_{k}E\left\{{\tilde{X}}_{k}V_{k}^{T}\right\}+E\left\{V_{k}{\tilde{X}}_{k}^{T}\right\}H_{k}^{T}+E\left\{V_{k}V_{k}^{T}\right\}\\ & =R_{k}-H_{k}P_{k}H_{k}^{T}. \end{array}$$

Then, the covariance of the difference sequence between innovation and residual can be determined based on the Theorem and Equations (18)–(19), namely,

(20) $$\begin{array}{ll} E\left[\left(\eta_{k}-\varepsilon_{k}\right){\left(\eta_{k}-\varepsilon_{k}\right)}^{T}\right] & =E\left(\varepsilon_{k}\varepsilon_{k}^{T}\right)+E\left(\eta_{k}\eta_{k}^{T}\right)-E\left(\varepsilon_{k}\eta_{k}^{T}\right)-E\left(\eta_{k}\varepsilon_{k}^{T}\right)\\ & \approx H_{k/k-1}P_{k/k-1}H_{k/k-1}^{T}+R_{k}+R_{k}-H_{k}P_{k}H_{k}^{T}-2\left(H_{k/k-1}P_{k}H_{k}^{T}-R_{k}K_{k}^{T}H_{k}^{T}+R_{k}\right)\\ & =H_{k/k-1}P_{k/k-1}H_{k/k-1}^{T}-H_{k}P_{k}H_{k}^{T}. \end{array}$$

Substituting for $P_{k/k-1}$ from Equation (4) into Equation (20), the covariance of the difference sequence can be rewritten as

(21) $$\begin{array}{ll} E\left[\left(\eta_{k}-\varepsilon_{k}\right){\left(\eta_{k}-\varepsilon_{k}\right)}^{T}\right] & =H_{k/k-1}P_{k/k-1}H_{k/k-1}^{T}-H_{k}P_{k}H_{k}^{T}\\ & =H_{k/k-1}\left[\sum_{i=0}^{2n}\omega_{i}^{\left(c\right)}\left(\chi_{k/k-1}^{\left(i\right)}-{\hat{X}}_{k/k-1}\right){\left(\chi_{k/k-1}^{\left(i\right)}-{\hat{X}}_{k/k-1}\right)}^{T}+\Gamma_{k-1}Q_{k-1}\Gamma_{k-1}^{T}\right]H_{k/k-1}^{T}\\ & -H_{k}P_{k}H_{k}^{T}. \end{array}$$

Then, it can be verified that

(22) $$\begin{array}{ll} H_{k/k-1}\Gamma_{k-1}Q_{k-1}\Gamma_{k-1}^{T}H_{k/k-1}^{T} & =E\left[\left(\eta_{k}-\varepsilon_{k}\right){\left(\eta_{k}-\varepsilon_{k}\right)}^{T}\right]\\ & -H_{k/k-1}\left[\sum_{i=0}^{2n}\omega_{i}^{\left(c\right)}\left(\chi_{k/k-1}^{\left(i\right)}-{\hat{X}}_{k/k-1}\right){\left(\chi_{k/k-1}^{\left(i\right)}-{\hat{X}}_{k/k-1}\right)}^{T}\right]H_{k/k-1}^{T}+H_{k}P_{k}H_{k}^{T}. \end{array}$$

On the other hand, the expectation of the difference sequence $E\left[\left(\eta_{k}-\varepsilon_{k}\right){\left(\eta_{k}-\varepsilon_{k}\right)}^{T}\right]$ can be approximated using a limited number of samples

(23) $$E\left[\left(\eta_{k}-\varepsilon_{k}\right){\left(\eta_{k}-\varepsilon_{k}\right)}^{T}\right]=\frac{1}{M}\sum_{j=1}^{M-1}\left(\eta_{k-j}-\varepsilon_{k-j}\right){\left(\eta_{k-j}-\varepsilon_{k-j}\right)}^{T}$$

where *M* is the window size. 

When the unknown elements in $Q_{k-1}$ is less than the rank of $H_{k/k-1}$, the unique solution can be obtained through Equation (22). Otherwise, some unknown elements in $Q_{k-1}$ can be assigned by their previous estimates. Additionally, $Q_{k-1}$ is normally a diagonal matrix. Therefore, the computational load can be further reduced. 

In the radar tracking system, the rank of $H_{k/k-1}$ is not less than the number of unknowns in $Q_{k-1}$. Thus, the condition for solving unique solutions is well satisfied. 

### 3.2. Adaptive **R** Estimation

In practical applications, the measurement noise covariance ***R*** is closely related to the performance of the radar. Due to different external and internal time varying disturbances, ***R*** is also time varying and should be estimated adaptively.

A relatively new method, RMNCE, used to estimate the measurement noise covariance can be applied to the systems with redundant measurements [33,34]. Assume that $Z_{1}\left(k\right)$ and $Z_{2}\left(k\right)$ are measurements of the true value $Z_{T}\left(k\right)$. Considering the steady-state and random error of the measurement, their expression yields

(24) $$\{\begin{array}{c} Z_{1}\left(k\right)=Z_{T}\left(k\right)+f_{1}\left(k\right)+V_{1}\left(k\right)\\ Z_{2}\left(k\right)=Z_{T}\left(k\right)+f_{2}\left(k\right)+V_{2}\left(k\right) \end{array}$$

where $f_{1}\left(k\right)$ and $f_{2}\left(k\right)$ are steady items of the measurement errors, $V_{1}\left(k\right)$ and $V_{2}\left(k\right)$ are uncorrelated, zero-mean Gaussian random noise.

When the measurement errors meet the following conditions:

(25) $$\{\begin{array}{c} \mathrm{diag}\left[\begin{array}{ccc} \cdots & {\left(f_{1}^{i}\left(k\right)-f_{1}^{i}\left(k-1\right)\right)}^{2} & \cdots \end{array}\right]\leq E\left\{V_{1}\left(k\right)V_{1}{\left(k\right)}^{T}\right\}\\ \mathrm{diag}\left[\begin{array}{ccc} \cdots & {\left(f_{2}^{i}\left(k\right)-f_{2}^{i}\left(k-1\right)\right)}^{2} & \cdots \end{array}\right]\leq E\left\{V_{2}\left(k\right)V_{2}{\left(k\right)}^{T}\right\} \end{array}$$

the covariance of the random noise for measurement $Z_{1}\left(k\right)$ and $Z_{2}\left(k\right)$ can be estimated as

(26) $$\left\{ \begin{array}{c} R_{1}=\frac{E\left[\nabla Z\left(k\right)\nabla Z{\left(k\right)}^{T}\right]+E\left[\Delta Z_{1}\left(k\right)\Delta Z_{1}{\left(k\right)}^{T}\right]-E\left[\Delta Z_{2}\left(k\right)\Delta Z_{2}{\left(k\right)}^{T}\right]}{4}\\ R_{2}=\frac{E\left[\nabla Z\left(k\right)\nabla Z{\left(k\right)}^{T}\right]-E\left[\Delta Z_{1}\left(k\right)\Delta Z_{1}{\left(k\right)}^{T}\right]+E\left[\Delta Z_{2}\left(k\right)\Delta Z_{2}{\left(k\right)}^{T}\right]}{4} \end{array}\right.$$

where

(27) $$\{\begin{array}{c} \begin{array}{l} \nabla Z\left(k\right)=\Delta Z_{1}\left(k\right)-\Delta Z_{2}\left(k\right)\\ \Delta Z_{1}\left(k\right)=Z_{1}\left(k\right)-Z_{1}\left(k-1\right) \end{array}\\ \Delta Z_{2}\left(k\right)=Z_{2}\left(k\right)-Z_{2}\left(k-1\right) \end{array}$$

The proof is shown in the Appendix. For a radar network, the radars can provide the range and azimuth measurements $Z_{k}$ by processing the reflected signal from the target. The measurement error can be classified into the steady-state error $f_{M}\left(k\right)$ and the random error $V_{R}\left(k\right)$ as follows:

(28) $$Z_{k}=Z_{T}\left(k\right)+f_{M}\left(k\right)+V_{R}\left(k\right).$$

Similarly, a redundant measurement $Z_{k}^{R}$ from the other radar node can be expressed as

(29) $$Z_{k}^{R}=Z_{T}\left(k\right)+f_{M}^{R}\left(k\right)+V_{R}^{R}\left(k\right)$$

where $f_{M}^{R}\left(k\right)$ denotes the steady-state error of the redundant measurement system, and $V_{R}^{R}\left(k\right)$ is the zero-mean white noise, which is uncorrelated with $V_{R}\left(k\right)$.

The steady-state errors of the (redundant) measurement are stable over a short period, so the difference between every two adjacent time steps of them can be neglected compared to the noise. Hence, the conditions in Equation (25) are well satisfied, and the measurement noise covariance can be estimated as:

(30) $$\left\{ \begin{array}{l} R_{k}=\left\{E\left[\nabla Z\left(k\right)\nabla Z{\left(k\right)}^{T}\right]+E\left[\left(Z_{k}-Z_{k-1}\right){\left(Z_{k}-Z_{k-1}\right)}^{T}\right]-E\left[\left(Z_{k}^{R}-Z_{k-1}^{R}\right){\left(Z_{k}^{R}-Z_{k-1}^{R}\right)}^{T}\right]\right\}/4\\ \nabla Z\left(k\right)=\left(Z_{k}-Z_{k-1}\right)-\left(Z_{k}^{R}-Z_{k-1}^{R}\right) \end{array}\right..$$

Considering the smoothness of the covariance estimation, a recursive estimation formula is used. Finally, the measurement noise covariance can be obtained as

(31) $$\left\{ \begin{array}{l} {\hat{R}}_{k}=\left(1-d_{k}\right)R_{k-1}+d_{k}R_{k}\\ d_{k}=\frac{1-b}{1-b^{k+1}} \end{array}\right..$$

where *b* is the fading factor, 0 < *b* < 1. 

### 3.3. Adaptive UKF Scheme

Based on the adaptive methods described above, the proposed adaptive UKF scheme aimed at target tracking in the presence of unknown time-varying noise covariance can be implemented as follows:

Step 1: Initialize the original estimated state value ${\hat{X}}_{0}$ and covariance $P_{0}$.

Step 2: Calculate the sigma points based on Equation (3).

Step 3: Apply the innovation and residual sequences to obtain the linear matrix Equation (22) and acquire ***Q*** by solving the equation.

Step 4: Calculate the state and measurement prediction according to Equations (4)–(6). 

Step 5: Use the raw measurement and redundant measurement sequences to estimate ***R*** via Equations (30) and (31).

Step 6: Calculate Kalman gain and filtering solutions through Equations (7) and (8).

Step 7: For the next sample, implement steps from 2 to 6.

## 4. Simulation Results and Discussion

In this section, the effectiveness of the proposed adaptive UKF algorithm for maneuvering target tracking will be illustrated through the simulations of different cases. 

### 4.1. Simulation Parameter and Cases

**Figure 1:**
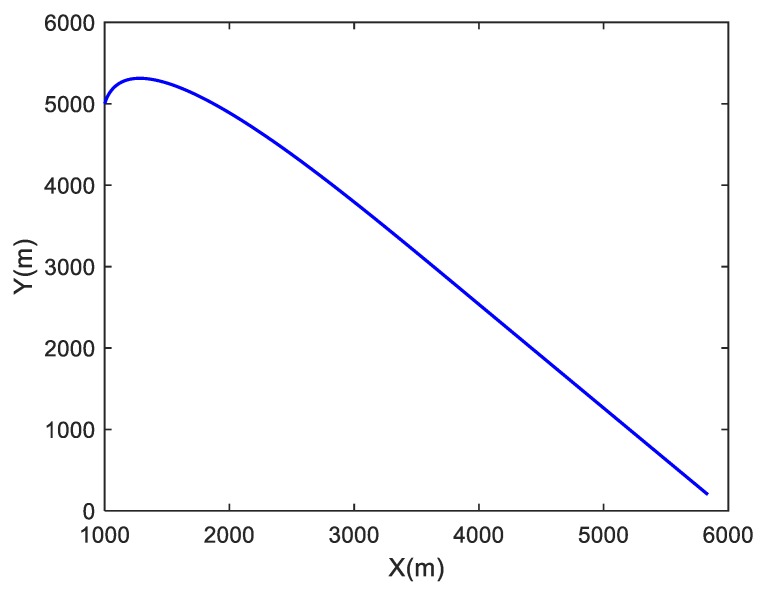
Simulated target trajectory.

**Figure 2:**
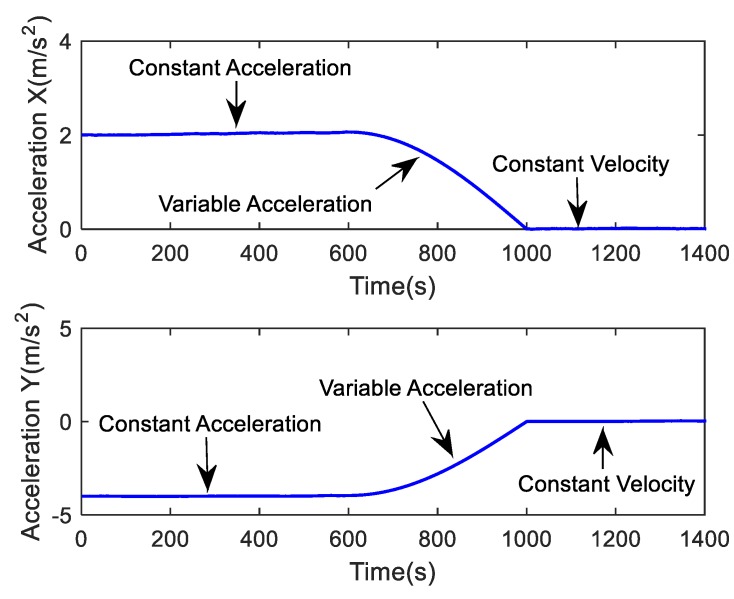
Actual curves of the acceleration.

The simulated trajectory considered in the simulation is in the *x*-*y* plane. It is assumed that the target makes a turn movement, then an approximate linear motion. The target conducts a constant-acceleration curvilinear motion during 0–600 s, a variable acceleration motion during 601–1000 s and a constant-velocity straight-line during 1001–1400 s. The initial coordinate of the target is $\left(x_{0},y_{0}\right)=\left(1000\;m,5000\;m\right)$, its initial velocity is $\left({\dot{x}}_{0},{\dot{y}}_{0}\right)=\left(10\;m/s,50\;m/s\right)$ and its initial acceleration is $\left({\ddot{x}}_{0},{\ddot{y}}_{0}\right)=\left(2\;m/s^{2},-4\;m/s^{2}\right)$. In the simulation, the process noise covariance matrix is set to be Q = diag[0.001 0.001]. A true target trajectory is depicted in Figure 1, and the actual curves of the acceleration are drawn in Figure 2.

As shown in Figure 2, the target performed a dynamic maneuver during the period from 601 s to 1000 s. Note that maneuver accelerations can lead to a mismatch in the system model on which the tracking filter relies. Therefore, dynamic maneuvers will cause potential changes to the process noise covariance.

**Simulation Case 1:** The measurement noise covariance matrix ***R*** = diag[100 0.001^2^] is known and the process noise covariance matrix ***Q*** varies over time. During the period 200–350 s, the process noise covariance matrix is assigned to be ***Q*** = diag[0.015 0.015].

**Simulation Case 2:** The measurement noise covariance matrix ***R*** is uncertain, and the process noise covariance matrix ***Q*** is known. The measurement noise covariance matrix is taken as ***R*** = diag[20 × 100 20 × 0.001^2^] during the period 200–350 s, and it is assigned to be ***R*** = diag[100 0.001^2^] for the remaining periods.

**Simulation Case 3:** Both the measurement noise covariance matrix ***R*** and the process noise covariance matrix ***Q*** are uncertain. In this case, the changes in Case 1 and Case 2 are implemented simultaneously.

In the filters, the target dynamic equation applied in different simulation cases is

(32) $$X_{k}=\left[\begin{array}{cccccc} 1 & 0 & T & 0 & T^{2}/2 & 0\\ 0 & 1 & 0 & T & 0 & T^{2}/2\\ 0 & 0 & 1 & 0 & T & 0\\ 0 & 0 & 0 & 1 & 0 & T\\ 0 & 0 & 0 & 0 & 1 & 0\\ 0 & 0 & 0 & 0 & 0 & 1 \end{array}\right]X_{k-1}+\Gamma_{k-1}W_{k-1}\begin{array}{cc} , & \Gamma_{k-1}=\left[\begin{array}{cc} T^{2}/2 & 0\\ 0 & T^{2}/2\\ T & 0\\ 0 & T\\ 1 & 0\\ 0 & 1 \end{array}\right] \end{array}$$

where $X_{k}=\left[\begin{array}{cccccc} x_{k} & y_{k} & {\dot{x}}_{k} & {\dot{y}}_{k} & {\ddot{x}}_{k} & {\ddot{y}}_{k} \end{array}\right]$. $\left(x_{k},y_{k}\right)$ denotes the position of the target at time *k*. $\left({\dot{x}}_{k},{\dot{y}}_{k}\right)$ and $\left({\ddot{x}}_{k},{\ddot{y}}_{k}\right)$ denote the velocity and acceleration of the target respectively. The sampling interval *T* is 1 s. The initial state ***X***_0_ = [1000 m, 5000 m, 10 m/s, 50 m/s, 2 m/s^2^, −4 m/s^2^], and the process noise covariance matrix is

(33) $$Q_{k-1}=\left[\begin{array}{cc} 0.001 & 0\\ 0 & 0.001 \end{array}\right].\;$$

The measurement systems are two radar observation stations. One is assumed to be located at the origin of the Cartesian coordinates and the other is regarded as the redundant measurement system, which can provide the same measurements of slant range $r_{k}$ and azimuth angle $\varphi_{k}$. The measurement model is expressed as

(34) $$Z_{k}=\left[\begin{array}{c} r_{k}\\ \varphi_{k} \end{array}\right]=\left[\begin{array}{c} \sqrt{x_{k}{}^{2}+y_{k}{}^{2}}\\ \arctan\left(\frac{y_{k}}{x_{k}}\right) \end{array}\right]+V_{k}.$$

The initial measurement noise covariance ***R*** = diag[100 0.001^2^]. The noise covariance of the redundant measurement is unknown, which can be estimated with the RMNCE algorithm.

### 4.2. Simulation Results

In view of the robustness and stability of the covariance matching and Sage-Husa adaptive schemes, only the process or measurement noise covariances are estimated by these methods in the first two Cases. For the third Case, a robust adaptive UKF scheme proposed in [38] is carried out as a contrast to our proposed method for estimating the process and measurement noise covariances simultaneously. The Q-estimation scheme in the robust adaptive UKF algorithm is the same as that applied in the adaptive fading UKF [25], which is used for comparison in the first Case. Furthermore, a new adaptive UKF proposed in [26], termed N-UKF, and an IMM algorithm constituted by two UKFs with different noise covariances, termed as IMM-UKF, are used for tracking the target in three different cases.

In all Cases, the simulations are run 100 times by utilizing the Monte Carlo method. The performances of the algorithms are assessed by the root mean square error of the position tracking, which is defined as

(35) $$E_{k}=\sqrt{\frac{1}{N}\sum_{i=1}^{N}\left[{\left({\hat{x}}_{k}^{i}-x_{k}\right)}^{2}+{\left({\hat{y}}_{k}^{i}-y_{k}\right)}^{2}\right]}$$

where *N* is the simulation times, $\left({\hat{x}}_{k}^{i},{\hat{y}}_{k}^{i}\right)$ denotes the filtering position of the target at time *k* in the *i*th simulation.

**Figure 3:**
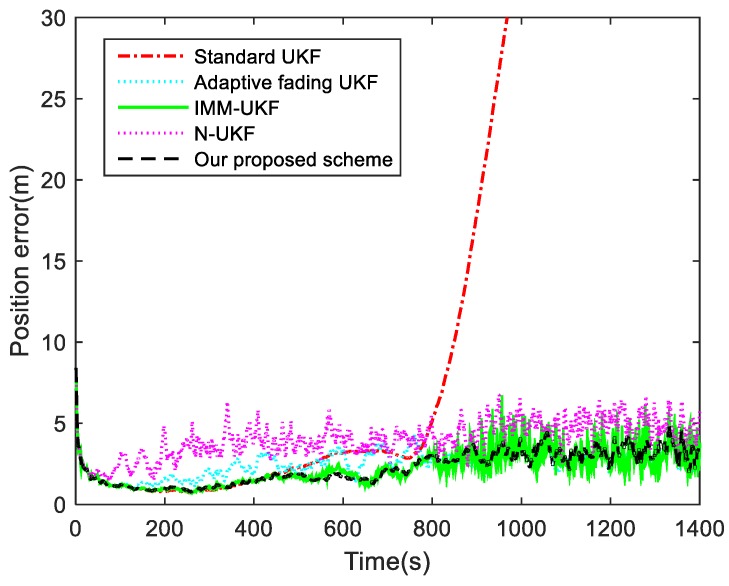
Position tracking errors for Case 1.

For the first Case, the position tracking errors of the standard UKF, adaptive fading UKF with covariance matching [25], IMM-UKF method, N-UKF algorithm, and our proposed Q-adaptive UKF are shown in Figure 3. The means and variances of the position tracking errors during the periods of 200–550 s and 550–1400 s are listed in Table 1.

As shown in Figure 3, it takes a longer time for the standard UKF to achieve the desired accuracy when the process noise covariance changes. A maneuver of the target for 400 s deteriorates the estimation of the standard UKF until the end of the simulation. The statistical errors of the standard UKF listed in Table 1 demonstrates that the potential process noise changes caused by target maneuver lead to an increase in the position errors, from 1.4549 m to 25.7565 m. For the adaptive fading UKF algorithm, in order to ensure that the process noise covariance does not change too much during the correction, the adaptive fading factor is limited in a certain range. Otherwise, the over-adjusted ***Q*** will lead to a divergence of the filter since the mismatches. Under the constraints, the position tracking error of the adaptive fading UKF algorithm is decreased compared with the standard UKF. Comparing the means and variances of the position errors during [200 s, 550 s] and [550 s, 1400 s] intervals, it is clear that the filtering performances of the used methods except for the standard UKF are all robust. As shown in Table 1, the tracking accuracy of our proposed Q-adaptive UKF scheme is almost the same as that of the IMM-UKF method, which demonstrates that both algorithms can resist the uncertainty of process noise. However, the computational load of IMM-UKF method is approximately two times higher than that of our proposed adaptive UKF scheme. In addition, although the N-UKF algorithm resists the disturbance of the changing statistics properties of states, its accuracy is not optimal due to the neglect of the correlativity between the innovation and residual sequences. In this case, the simulation results demonstrate that our proposed method is affected by neither the time-varying process noise nor the maneuvering motion models.

**Figure 4:**
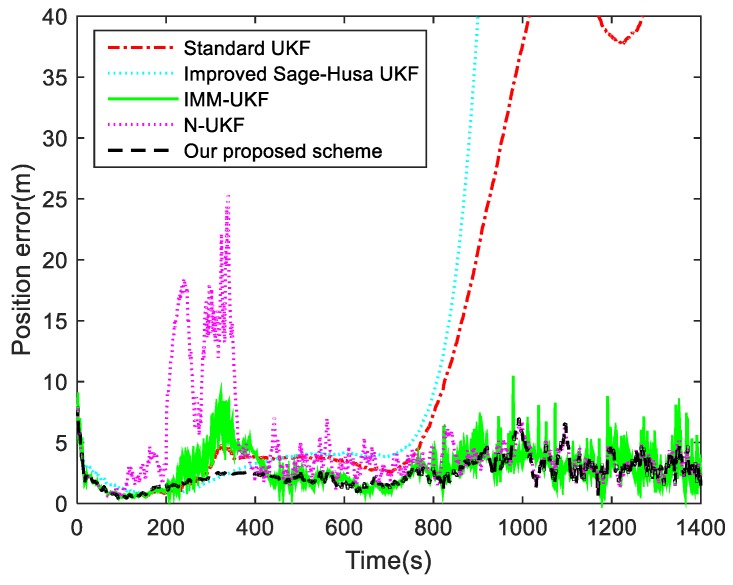
Position tracking errors for Case 2.

**Figure 5:**
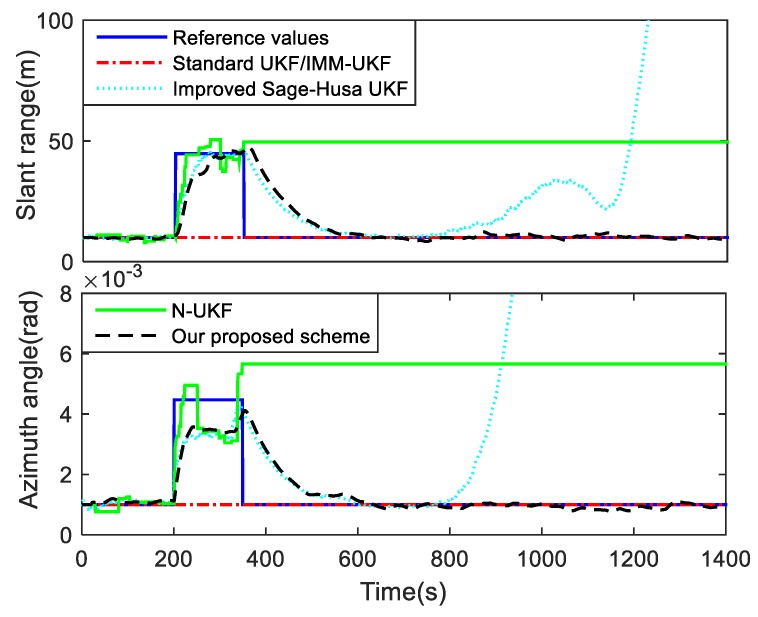
Estimated measurement noise standard deviations for Case 2.

For the second Case, in order to verify the adaptive performance of our proposed UKF, the improved Sage-Husa adaptive algorithm in [31] is introduced to the UKF algorithm for target tracking. Meanwhile, contrast simulations of the standard UKF, IMM-UKF method and N-UKF algorithm are conducted in this case. The position tracking errors of these algorithms are shown in Figure 4, and the means and variances of the position tracking errors during the periods of 200–350 s and 601–1400 s are listed in Table 2. The measurement noise standard deviations used in these algorithms are shown in Figure 5.

It can be seen from Figure 4 and Table 2 that the performances of the standard UKF and IMM-UKF methods deteriorate when the measurement noise changes during the period of 200–350 s. As shown in Figure 5, the measurement noise standard deviations used in the standard UKF and IMM-UKF method are fixed values, which will be mismatched when the noise changes. Furthermore, due to the mismatched system model, the position tracking error of the standard UKF increases significantly after the 600th second. Although the improved Sage-Husa UKF algorithm can overcome the time-varying noise covariance of the measurement, it diverges when the system model changes. It can be found in Figure 5 that when the target performs a maneuvering motion, the measurement noise standard deviations estimated by the improved Sage-Husa UKF algorithm are biased because the coupled innovation is contaminated. The N-UKF algorithm can effectively detect the filtering divergence when the noise variances increase. However, due to the negative influence of the inaccurate estimates of the process noise covariance, the theoretical estimate error may be more than the actual estimation error, and thus when the noise variance decreases the detection will fail and the measurement noise standard deviations are not updated. By contrast, our proposed R-adaptive UKF is immune to the state estimation and can modify the measurement noise covariance effectively. When the measurement noise changes, both our proposed method and the improved Sage-Husa UKF algorithm require a delay to match the actual noise variances. This is because the estimate covariances are calculated cumulatively based on the data in a sliding window. The fading factor and the window size are usually selected by experience as they make a trade-off between the smoothness and rapidity of the measurement noise covariance estimation. In our simulations, the fading factor is 0.98 and the window size for estimation is chosen as 25. As expected, the proposed R-adaptive scheme avoids the divergence occurred in the improved Sage-Husa algorithm. In addition, the position errors of the standard UKF and the improved Sage-Husa UKF algorithm grow to 40 m without the Q-estimation, which means that the adaptive process noise covariance in our proposed scheme can contribute to the increase in the tracking precision.

**Figure 6:**
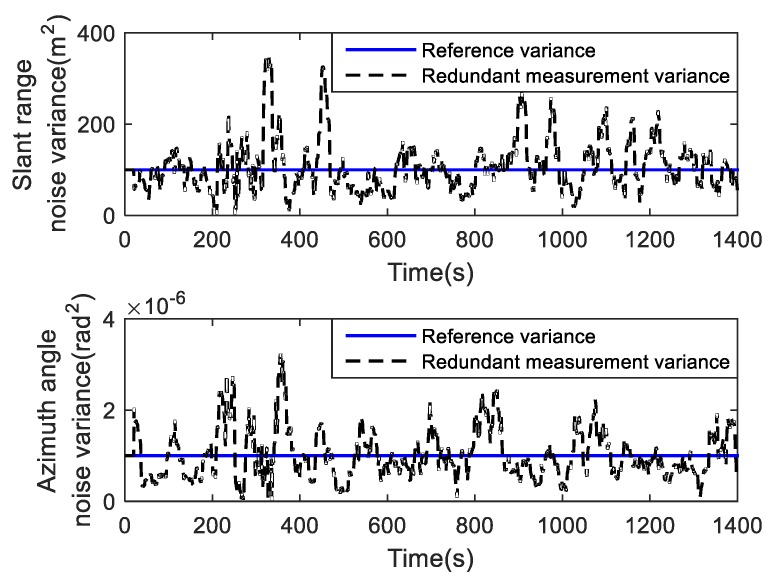
Estimated redundant measurement noise variance for Case 2.

Figure 6 shows the estimated results of the redundant measurement noise variance. It can be seen that the estimate variances fluctuate around the reference values. To further evaluate the performance of the estimation, the means of the estimated noise variance of the range and azimuth are calculated. Their results are 100.8194 m^2^ and 1.0507 × 10^−6^ rad^2^, and the reference variances are 100 m^2^ and 1.0 × 10^−6^ rad^2^. It is clear that the RMNEC algorithm can provide a reliable estimation for the redundant measurement variances.

**Figure 7:**
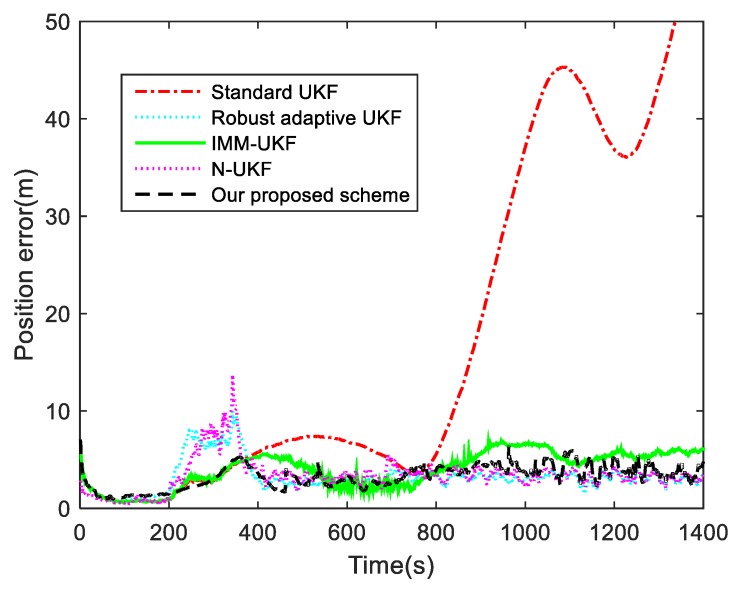
Position tracking errors for Case 3.

For the third Case, the performance and feasibility of our proposed adaptive UKF scheme are tested when Q and R change simultaneously. In this case, the standard UKF, IMM-UKF method, N-UKF algorithm and a robust adaptive UKF scheme in [38] are applied to tracking the target. The contrast results of the filtering position errors are presented in Figure 7. The means and variances of the position tracking errors during the periods of 200–550 s and 550–1400 s are listed in Table 3.

As shown in Figure 7 and Table 3, the filtering result of the standard UKF algorithm becomes inaccurate in the presence of the process and measurement noise covariance variations. The time-varying noise covariances lead to the divergence of the standard UKF, even though the noise covariances return to its priori value and changes disappear after the 350th second. This is because the standard UKF algorithm has no adaptive abilities. As described in Section 2.2, the contaminated noise covariances can influence the filtering gain and estimation covariances, which would cause the filtering divergence. The robust adaptive UKF and the N-UKF algorithm can both avoid the filtering divergence, but the performances of the Q-matching method in these algorithms are affected by the varying measurement noise covariance. When the process and measurement noise covariances changed simultaneously, it is hard to distinguish the type of the fault (either measurement interference or process noise uncertainty) only though the statistical information of the innovation. Hence, the adaptation procedures in both the robust adaptive UKF and the N-UKF algorithm failed to accord with the noise changes. During the period of 601–1000 s, the fault was detected and isolated effectively by the robust adaptive UKF and N-UKF algorithm when only the process noise covariance changed, which reduced the position tracking error. Compared with our proposed scheme, the computational load of the IMM-UKF method is doubled, while the filtering results of IMM-UKF are also not optimal. One reason for this unstable performance is that the models and the switching probabilities in the IMM-UKF method are chosen by experience, and furthermore, the framework of the IMM method is designed for uncertain system models. In our schemes, the changing measurement noise covariance is estimated through the redundant measurements, which are entirely immune to the state estimation. This means that the process noise covariance can be estimated depending on the “clean” innovation and residual sequences, which have considered the influence of the contaminated measurement noise. Thus, our proposed algorithm still maintained good tracking accuracy when the process and measurement noise covariance varied during the period of 200–350 s. The simulation results prove that our proposed adaptive UKF scheme with Q and R-adaptive can achieve accurate estimation and meet the requirements of target tracking.

### 4.3. Discussion

The adaptive filtering problems for time-varying noise covariances involved in nonlinear target tracking systems have been researched, and an innovative adaptive UKF scheme has been developed to improve the tracking accuracy and stability. From the simulation results in Figure 3 and Table 1, it is obvious that after the adaptive processes, the divergence of the standard UKF has been effectively suppressed. However, it should be noted that the process noise covariance solution is likely to be negative when resolving the linear matrix Equation (22) because of the limited size of window in Equation (23) and the measurement approximation errors. Therefore, in order to avoid such situations, an absolute or scale operator should be applied to the covariance solution in practical applications [27,39].

Moreover, the varying measurement noise covariance also has a great influence on the filtering result, which is shown in Figure 4 and Table 2. Although our proposed R-adaptive scheme can suppress the noise and avoid the divergence which often occurs in the improved Sage-Husa method, it relies on a redundant measurement system. With the absence of the redundant measurements, the RMNCE method would be infeasible. In this situation, if the computational power permits, an alternative scheme named improved second order mutual difference estimation can be applied to deal with the single measurement noise covariance estimation problem [40].

When the process noise and measurement noise needed to be estimated simultaneously, the filtering accuracy was well maintained by applying the RMNCE method and tuning the process noise covariance adaptively based on the correct correlation of the innovation and residual sequences. Although our proposed adaptive schemes are used for UKF, it can also be applied for EKF, since no special feature of UKF is used in estimating Q or R. Furthermore, the proposed correction schemes avoid the negative impact of the process noise on estimating the measurement noise covariance. It can be seen from the target tracking simulation results, as shown in the simulation results in Figure 7 and Table 3, that our proposed adaptive scheme can solve the uncertainties of the noise covariance and make a considerable contribution to the filtering accuracy and stability.

In summary, the proposed adaptive UKF scheme can provide accuracy and reliable tracking in challenging environments, compared with the standard UKF, IMM-UKF method and the current adaptive UKF strategies. The next step is to broaden the application fields of the proposed adaptive scheme, and further extend the estimation of measurement noise covariance to a single measurement system.

## 5. Conclusions

Accurate estimation of the dynamic parameters in the maneuvering target relies in the performance of the filter. However, the standard and current adaptive UKF algorithms will diverge whenever the filtering models involve the time-varying noise covariance. To improve the stability and accuracy of the target tracking, a new adaptive UKF algorithm is proposed. In the proposed method, the covariance of the process and measurement noise is tuned in real time by using the innovation, residual and redundant measurement sequences. The process noise covariance can be obtained by resolving the linear matrix equation, which is deduced from the expectation of the difference sequence between innovation and residual. The measurement noise covariance is estimated through the RMNCE method by using the redundant measurement from the multi radar system. Simulation results demonstrate that the adaptive UKF scheme presented in this paper can effectively restrain the filtering divergence and has a better filtering performance compared with the standard and existing adaptive UKF algorithms. For the future, the influence of the correlation between the measurements is worth further research, which will benefit the accuracy of the measurement noise covariance estimation. In another way, the modern artificial intelligence methods may avoid the dilemma of the filtering noise covariance estimation.

## Figures and Tables

**Table 1:** Position errors of the different schemes for Case 1.

Algorithm	200–550 s		550–1400 s	
	Mean (m)	Variance (m^2^)	Mean (m)	Variance (m^2^)
Standard UKF	1.4549	0.3783	25.7565	341.6688
Adaptive fading UKF	2.1264	0.2519	2.8608	0.2411
IMM-UKF	1.3258	0.1141	2.7311	0.5510
N-UKF	3.7332	0.4344	4.5229	0.6996
Our proposed scheme	1.3398	0.1080	2.7165	0.5497

**Table 2:** Position errors of the different schemes for Case 2.

Algorithm	200–350 s		600–1400 s	
	Mean (m)	Variance (m^2^)	Mean (m)	Variance (m^2^)
Standard UKF	2.9130	0.7260	26.7439	337.3589
Improved Sage-Husa UKF	2.4260	0.8123	359.2692	2.1492 × 10^5^
IMM-UKF	3.0745	2.2106	2.8958	1.3549
N-UKF	7.9900	27.0631	3.5731	0.9831
Our proposed scheme	1.9730	0.1264	2.9107	1.0564

**Table 3:** Position errors of the different schemes for Case 3.

Algorithm	200–550 s		550–1400 s	
	Mean (m)	Variance (m^2^)	Mean (m)	Variance (m^2^)
Standard UKF	4.8845	4.0442	26.8136	316.1329
Robust adaptive UKF	4.6399	5.0780	3.0834	0.2204
IMM-UKF	3.9900	1.3483	4.6487	2.3042
N-UKF	4.7748	5.6900	3.3517	0.3042
Our proposed scheme	3.0623	1.0426	3.7313	0.7709

## Appendix

This Appendix gives the proof of noise covariance estimation based on the redundant measurement. As shown in Equation (24), the measurements from different systems are available. In this condition, the first order difference sequence of two measurement systems can be calculated as

(A1) $$\left\{ \begin{array}{ll} \Delta Z_{1}\left(k\right) & =Z_{1}\left(k\right)-Z_{1}\left(k-1\right)=\left[Z_{T}\left(k\right)+f_{1}\left(k\right)+V_{1}\left(k\right)\right]-\left[Z_{T}\left(k-1\right)+f_{1}\left(k-1\right)+V_{1}\left(k-1\right)\right]\\ & =\left[Z_{T}\left(k\right)-Z_{T}\left(k-1\right)\right]+\left[f_{1}\left(k\right)-f_{1}\left(k-1\right)\right]+\left[V_{1}\left(k\right)-V_{1}\left(k-1\right)\right]\\ \Delta Z_{2}\left(k\right) & =Z_{2}\left(k\right)-Z_{2}\left(k-1\right)=\left[Z_{T}\left(k\right)+f_{2}\left(k\right)+V_{2}\left(k\right)\right]-\left[Z_{T}\left(k-1\right)+f_{2}\left(k-1\right)+V_{2}\left(k-1\right)\right]\\ & =\left[Z_{T}\left(k\right)-Z_{T}\left(k-1\right)\right]+\left[f_{2}\left(k\right)-f_{2}\left(k-1\right)\right]+\left[V_{2}\left(k\right)-V_{2}\left(k-1\right)\right] \end{array}\right.$$

Then, the second-order mutual difference sequence of the measurements yields

(A2) $$\begin{array}{ll} \nabla Z\left(k\right) & =\Delta Z_{1}\left(k\right)-\Delta Z_{2}\left(k\right)\\ & =\left[f_{1}\left(k\right)-f_{1}\left(k-1\right)\right]-\left[f_{2}\left(k\right)-f_{2}\left(k-1\right)\right]+\left[V_{1}\left(k\right)-V_{1}\left(k-1\right)\right]-\left[V_{2}\left(k\right)-V_{2}\left(k-1\right)\right]. \end{array}$$

When the condition in Equation (25) is well satisfied and consider $V_{1}\left(k\right)$ and $V_{2}\left(k\right)$ are uncorrelated, zero-mean Gaussian random noise, the autocorrelation of the first order difference sequences can be obtained as

(A3) $$\begin{array}{ll} E\left[\Delta Z_{1}\left(k\right)\Delta Z_{1}{\left(k\right)}^{T}\right] & =E\left\{\left[Z_{1}\left(k\right)-Z_{1}\left(k-1\right)\right]{\left[Z_{1}\left(k\right)-Z_{1}\left(k-1\right)\right]}^{T}\right\}\\ & =\left[Z_{T}\left(k\right)-Z_{T}\left(k-1\right)\right]{\left[Z_{T}\left(k\right)-Z_{T}\left(k-1\right)\right]}^{T}\\ & +\mathrm{diag}\left[\begin{array}{ccc} \cdots & {\left(f_{1}^{i}\left(k\right)-f_{1}^{i}\left(k-1\right)\right)}^{2} & \cdots \end{array}\right]+E\left\{V_{1}\left(k\right)V_{1}{\left(k\right)}^{T}\right\}\\ & \approx \left[Z_{T}\left(k\right)-Z_{T}\left(k-1\right)\right]{\left[Z_{T}\left(k\right)-Z_{T}\left(k-1\right)\right]}^{T}+E\left\{V_{1}\left(k\right)V_{1}{\left(k\right)}^{T}\right\}\\ & =\left[Z_{T}\left(k\right)-Z_{T}\left(k-1\right)\right]{\left[Z_{T}\left(k\right)-Z_{T}\left(k-1\right)\right]}^{T}+2R_{1}. \end{array}$$

Similarly,

(A4) $$\begin{array}{ll} E\left[\Delta Z_{2}\left(k\right)\Delta Z_{2}{\left(k\right)}^{T}\right] & =E\left\{\left[Z_{2}\left(k\right)-Z_{2}\left(k-1\right)\right]{\left[Z_{2}\left(k\right)-Z_{2}\left(k-1\right)\right]}^{T}\right\}\\ & =\left[Z_{T}\left(k\right)-Z_{T}\left(k-1\right)\right]{\left[Z_{T}\left(k\right)-Z_{T}\left(k-1\right)\right]}^{T}\\ & +\mathrm{diag}\left[\begin{array}{ccc} \cdots & {\left(f_{2}^{i}\left(k\right)-f_{2}^{i}\left(k-1\right)\right)}^{2} & \cdots \end{array}\right]+E\left\{V_{2}\left(k\right)V_{2}{\left(k\right)}^{T}\right\}\\ & \approx \left[Z_{T}\left(k\right)-Z_{T}\left(k-1\right)\right]{\left[Z_{T}\left(k\right)-Z_{T}\left(k-1\right)\right]}^{T}+E\left\{V_{2}\left(k\right)V_{2}{\left(k\right)}^{T}\right\}\\ & =\left[Z_{T}\left(k\right)-Z_{T}\left(k-1\right)\right]{\left[Z_{T}\left(k\right)-Z_{T}\left(k-1\right)\right]}^{T}+2R_{2}. \end{array}$$

Then, we can obtain that

(A5) $$E\left[\Delta Z_{1}\left(k\right)\Delta Z_{1}{\left(k\right)}^{T}\right]-E\left[\Delta Z_{2}\left(k\right)\Delta Z_{2}{\left(k\right)}^{T}\right]=2R_{1}-2R_{2}.$$

On the other hand, the autocorrelation of the second-order difference sequences can be obtained as

(A6) $$\begin{array}{ll} E\left[\nabla Z\left(k\right)\nabla Z{\left(k\right)}^{T}\right] & =E\left\{\left[\Delta Z_{1}\left(k\right)-\Delta Z_{2}\left(k\right)\right]{\left[\Delta Z_{1}\left(k\right)-\Delta Z_{2}\left(k\right)\right]}^{T}\right\}\\ & =\mathrm{diag}\left[\begin{array}{ccc} \cdots & {\left(f_{1}^{i}\left(k\right)-f_{1}^{i}\left(k-1\right)\right)}^{2} & \cdots \end{array}\right]+E\left\{V_{1}\left(k\right)V_{1}{\left(k\right)}^{T}\right\}\\ & +\mathrm{diag}\left[\begin{array}{ccc} \cdots & {\left(f_{2}^{i}\left(k\right)-f_{2}^{i}\left(k-1\right)\right)}^{2} & \cdots \end{array}\right]+E\left\{V_{2}\left(k\right)V_{2}{\left(k\right)}^{T}\right\}\\ & +E\left\{V_{1}\left(k-1\right)V_{1}{\left(k-1\right)}^{T}\right\}+E\left\{V_{2}\left(k-1\right)V_{2}{\left(k-1\right)}^{T}\right\}\\ & \approx E\left\{V_{1}\left(k\right)V_{1}{\left(k\right)}^{T}\right\}+E\left\{V_{1}\left(k-1\right)V_{1}{\left(k-1\right)}^{T}\right\}\\ & +E\left\{V_{2}\left(k\right)V_{2}{\left(k\right)}^{T}\right\}+E\left\{V_{2}\left(k-1\right)V_{2}{\left(k-1\right)}^{T}\right\}\\ & =2R_{1}+2R_{2}. \end{array}$$

Finally, the random noise covariances of measurement for $Z_{1}\left(k\right)$ and $Z_{2}\left(k\right)$ can be estimated by solving Equations (A5) and (A6) as

(A7) $$\left\{ \begin{array}{c} R_{1}=\frac{E\left[\nabla Z\left(k\right)\nabla Z{\left(k\right)}^{T}\right]+E\left[\Delta Z_{1}\left(k\right)\Delta Z_{1}{\left(k\right)}^{T}\right]-E\left[\Delta Z_{2}\left(k\right)\Delta Z_{2}{\left(k\right)}^{T}\right]}{4}\\ R_{2}=\frac{E\left[\nabla Z\left(k\right)\nabla Z{\left(k\right)}^{T}\right]-E\left[\Delta Z_{1}\left(k\right)\Delta Z_{1}{\left(k\right)}^{T}\right]+E\left[\Delta Z_{2}\left(k\right)\Delta Z_{2}{\left(k\right)}^{T}\right]}{4} \end{array}\right.$$

This completes the proof. □
